# Incarceration and Perforation of a Sliding Hiatus Hernia: Report of a Case

**DOI:** 10.4021/gr319w

**Published:** 2011-09-20

**Authors:** Jody Parker, Sivakurmaran Sabanathan

**Affiliations:** a Department of Surgery, Ysbyty Gwynedd, Betsi Cadwaladr University Health Board, North Wales, UK

**Keywords:** Sliding hiatus hernia, Incarceration, Perforation

## Abstract

The potentially serious complications of paraoesophageal hiatus hernias are known but its counterpart the sliding hernia, is thought to be more benign in nature. We describe a 72 year old female admitted with epigastric pain after gorging on her Christmas meal, who proved a diagnostic difficulty for both the medical and surgical registrars and was found to have a perforated incarcerated sliding hiatus hernia on CT scan. A transhiatal oesophagectomy was performed as laparotomy findings confirmed a gangrenous perforated stomach and a gangrenous lower oesophagus. She recovered fully from the operation and is well to date. This case provides evidence that sliding hernias can cause serious complications and may be difficult to differentiate from other cardiovascular and abdominal pathologies. A high index of suspicion is required by medical professionals treating chest and epigastric pain.

## Introduction

Hiatus hernias are a common pathology and 95% of them are sliding whereby the oesophagus is shortened and the gastro oesophageal junction and part of the stomach are displaced into the chest through the hiatus of the diaphragm. The remainders are mostly paraoesophageal hernias where a portion of the stomach herniates into the chest adjacent to the oesophagus but the gastro-oesophageal junction remains within the abdomen. Many are asymptomatic especially the sliding variety, but presenting features can include reflux, regurgitation, postprandial breathlessness, early satiety and dysphagia especially in larger hernias. Most are identified during upper gastrointestinal endoscopy. Large paraoesophageal hernias can present acutely with gangrene or rupture of the intrathoracic stomach and are therefore repaired electively if the patient is willing and clinically fit [1]. Treatment otherwise involves symptomatic management with surgery as a final option.

Although the rarer variant, there are several reports in the literature of incarcerated, strangulated or ulcerated paraoesophageal hernias [1-4]. This however does not extend to the apparently benign but more common, sliding hernia. Ours is the first in the literature to be reported that we know of.

Even though incarcerated hiatus hernias are rare, it is important to consider them as a differential diagnosis for many surgical and medical presentations. The consequence of a delayed or missed diagnosis may be disastrous and the possible alternatives can be equally as important. These include acute coronary syndromes, acute respiratory pathology, intestinal perforation, aortic rupture or dissection and pancreatitis. Chest X-ray can provide clues such as left sided pulmonary effusion, visible large hernia above the diaphragm and pneumoperitoneum or mediastinum [3, 5]. Given the potential heterogeneity in both the presentation and interpretation of the assessing physician, liaising of both medical and surgical specialities is paramount. Traditionally an emergency laparotomy is performed but hiatus hernias are more frequently repaired electively and as an emergency through laparoscopic techniques [6].

## Case Report

We report a 72 year old lady who reported to the Emergency Department in an early evening of December with a six hour history of severe epigastric pain after a Christmas meal. The pain radiated to the back, was worse on breathing and associated with several episodes of vomiting. Past medical history included a hiatus hernia, idiopathic leg oedema (treated with bendroflumethiazide), osteoporosis and previous appendicectomy. Alcohol intake was approximately seven units weekly and she was a non-smoker.

**Figure 1:**
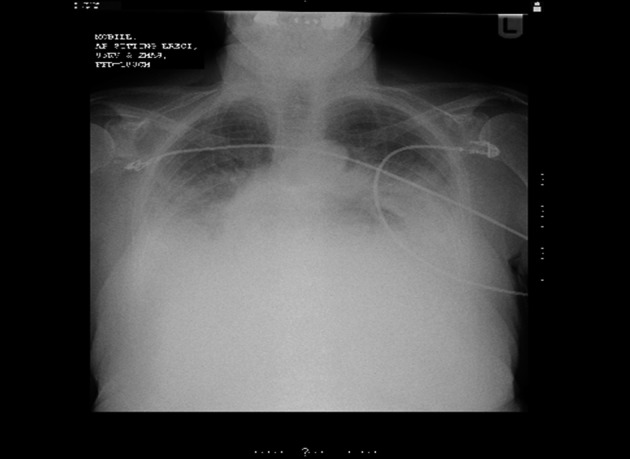
Chest X-ray taken on presentation to the Emergency Department. There is bilateral basal consolidation but no obvious hernia or pneumoperitoneum.

On examination her blood pressure was 103/60 mmHg, pulse rate 70 per minute, temperature 36 °C and respiratory rate 22 per minute. Heart sounds were normal but there were bilateral inspiratory basal crepitations on respiratory examination. Abdominal examination revealed epigastric tenderness with some voluntary guarding but no rebound or rigidity. Bowel sounds were present and normal. Initial investigations were performed including ECG, routine bloods including amylase and troponins, arterial blood gas and lactate. All tests were within normal limits except for a lactate of 3.7 (normal range 0.5-2.2 mmol/L) and marginally raised amylase of 170 (normal range < 120 U/L). Chest X-ray revealed bibasal consolidation but with no air under the diaphragm visible (Fig. 1) and abdominal X-ray showed no diagnostic features.

The Emergency Department doctors made referrals to both the on call medical and surgical teams. Surgeons were the first to review who interpreted there being no acute surgical pathology but advised to repeat the chest X-ray to ensure there was no unidentified pneumoperitoneum. Medical review added bronchial breathing to the examination findings and considered a range of diagnoses including cardiac failure, bronchopneumonia, intestinal perforation or pancreatitis. Repeat X-ray was performed later in the evening which did not reveal any new changes. Fluid balance monitoring was initiated and she was treated for the possible medical diagnoses with intravenous furosemide and broad spectrum antibiotics by the medical team.

**Figure 2:**
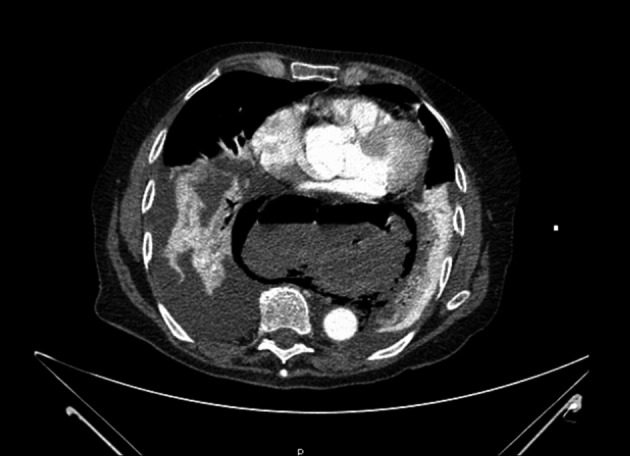
CT images taken after admission. The large hernia within the mediastinum is visible with surrounding air.

Throughout this time, there was detailed discussion regarding the potential diagnoses and the best place for this patient’s treatment to continue. The on call Emergency Department Consultant was called into to resolve the situation. Considering the diagnostic uncertainty and potentially serious pathologies, he arranged a CT scan to clarify (Fig. 2). This reported a large sliding hiatus hernia with a narrow neck. There was gas but no liquid identified around the hernia in the mediastinum and associated bilateral pleural effusions and consolidation. This confirmed the diagnosis of an incarcerated and ischaemic sliding hiatus hernia.

She was taken to theatre for a laparotomy during the early hours of the following morning. This showed that the upper one third of the stomach was within the sliding hernia which along with the distal half of the oesophagus, was gangrenous. There was a perforation of the stomach within this hernia with food debris within the sac. The remaining lower two thirds of the stomach although not within the hernia, were congested. In view of the distal gangrenous oesophagus, a transhiatal oesophagectomy was performed. A stomach tube was created and the oesophagogastric anastomosis lay within the neck.

Her recovery began on ITU where she spent six days in total. She received treatment for sepsis in addition to total parenteral nutrition. Bilateral chest drains were sited. A further five days were spent on high dependency before she was discharged to the surgical ward for ten days. Prior to discharge to a community rehabilitation unit, hoarseness of the voice was noted. This was followed up as an outpatient with ENT where a paralysed left vocal cord was diagnosed. Voice therapy was commenced and on review two months later in the surgical clinic, her voice had returned to normal. Intermittent dysphagia had been a problem since discharge for which a gastroscopy was arranged showing no stricture or other new pathology.

## Discussion

As this is the first case describing an incarcerated, perforated sliding hiatus hernia, we are unable to compare our presentation with others. Acute chest and epigastric pain can account for a large percentage of emergency department reviews. The main presenting features of this case were vomiting and acute epigastric pain following a large meal, which seem to be similar to the presenting features described for incarcerated or perforated paraoesophageal hernias [2-4]. Other presenting symptoms include acute chest pain, belching and dyspnoea [3, 4]. Risk factors for perforation in paraoesophageal hernias include size, increased age and incarceration [7]. All physicians dealing with such presentations should be aware of this diagnosis.

Diagnostic difficulties are evident when it comes to epigastric pain with a range of cardiothoracic and intra-abdominal pathologies being possible. A perforated hiatus hernia is rarely identified as a differential. Routine bloods including cardiac enzymes and amylase, blood gases including lactate and ECG can all help rule out differential diagnoses. Chest X-ray may provide the first clues but in our case the chest X-ray provided no diagnostic certainty in that there was no pneumoperitoneum or visible hernia and pleural effusions which are typically left sided, were bilateral. In our case the CT findings made the diagnosis clear and can be very important in establishing the diagnosis when a clinician is aware of the gravity of the presentation, but is unsure of the diagnosis and subsequent management plan.

It has been known for such cases to be treated as acute coronary syndromes with the usual anticoagulants resulting in subsequent marked operative implications when the real diagnosis is identified [4]. Treatment is in most cases by laparotomy and proceeding as necessary depending on the findings. Although some recommend that paraoesophageal hernias should be repaired electively due to the potential acute complications, the need for surgical intervention is debatable as the annual incidence of surgery for life threatening complications is still low [8]. Conversely sliding hernias are usually treated symptomatically as until now, serious complications were unheard of. This is an extremely rare complication of a common condition but she probably had a tight hiatus which caused the food to impact in the hernia. Patients with this abnormality should be advised to eat slowly and chew their food well. They should also avoid large volume meals. This followed a Christmas dinner.

In conclusion this case highlights the first serious complication of a sliding hiatus hernia and the need for both surgeons and medics to be aware and consider the diagnosis in patients with symptoms such as chest pain, epigastric pain, vomiting and dyspnoea.
